# Body mass index trajectories and age at menopause in a British birth cohort

**DOI:** 10.1016/j.maturitas.2008.02.009

**Published:** 2008-04-20

**Authors:** Rebecca Hardy, Gita D. Mishra, Diana Kuh

**Affiliations:** Medical Research Council Unit for Lifelong Health and Ageing, Department of Epidemiology and Public Health, Royal Free and University College London Medical School, 1-19 Torrington Place, London WC1E 6BT, United Kingdom

**Keywords:** Age at menopause, Underweight, BMI trajectories, Hormone therapy, Cohort study

## Abstract

**Objective:**

This study investigates the influence of body mass index (BMI) at ages 15, 20, 26, 36, and 43, and of BMI trajectories from 20 to 36 years on the timing of menopause and hormone therapy (HT) use until age 57 years.

**Methods:**

A nationally representative British cohort of 1583 women born in March 1946 with prospective data across the life course.

**Results:**

By age 57, a total of 695 women had experienced natural menopause while 431 women had started HT prior to menopause. Cox regression models indicated no significant associations between BMI at any age, or BMI trajectory, and timing of natural menopause. At every age BMI was strongly (*p* ≤ 0.01) and linearly associated with age at HT use and BMI from 26 years onwards was associated with age at first event (menopause or HT use). Decreasing BMI was associated with earlier HT use at all ages. These associations were not accounted for by parity, cigarette smoking or childhood and adult social class.

**Conclusion:**

BMI across the reproductive lifespan did not influence age at menopause to an extent that would be clinically relevant for postmenopausal health. Lower BMI at all ages and underweight trajectory were related to an earlier start of HT. Further studies are required to understand whether such relationships are due to underweight women experiencing menopause earlier (and because of menopausal symptoms starting HT earlier) than heavier women, or having behavioural characteristics related to earlier HT use, independent of menopause.

## 1 Introduction

Age at menopause has implications for the subsequent health of women. Late menopause has been linked to an increased risk of breast cancer [1], while early menopause is associated with increased risk of osteoporosis [2] and, with less consistent evidence, of cardiovascular disease [3,4] and with earlier cognitive decline [5–7]. Few adult environmental risk factors for age at menopause have been identified. Only cigarette smoking and nulliparity have consistently been associated with earlier age at natural menopause [8–12]. Body size and fat distribution have been considered in relation to age at menopause, as it is hypothesised that increased peripheral production of estrone in obese women might contribute to a delay in their menopause [13]. Results from general population samples have been inconsistent showing either no association [8–11,14–20] or that heavier women have a later menopause [12,21–25]. Further studies have considered body size as a consequence of menopause [26–28]. In cross-sectional studies it is impossible to ascertain the direction of any effect, while most previous longitudinal studies have collected just a single midlife premenopausal measure of body size, often measured only a few years prior to menopause [12,14,16,19,24,25].

It has been suggested that hormonal imbalances occurring as a result of weight change across the life course increase the rate of follicular atresia [29]. Hence, it may be weight change rather than weight *per se* that influences age at menopause. The few studies that have considered measures of body size in early adult life or change in size have mostly relied on recall of early adult weight [8,18,20,23,29].

Parity may be an explanation for any association between BMI trajectory across adult life and age at menopause. Nulliparous women have an earlier age at menopause than parous women in most studies [8–12] and tend to have lower BMI [30–32]. Subfertility, of which nulliparity may be a marker, is related to being obese as well as underweight [33] and subfertility may be a risk factor for early menopause. The only study to investigate specific measures of subfertility (other than nulliparity) and age at menopause found an association between early menopause and reporting a miscarriage, consulting a physician for fertility problems and having an interval of 5 or more years between the birth of their first and second child [34]. Hence, if the association between BMI and age at menopause reflects underlying fertility, women who have been obese as well as those who have been underweight throughout life may have earlier menopause than women who remain of normal weight or who gain weight over the life course.

Using data from the Medical Research Council (MRC) National Survey of Health and Development (NSHD) we will consider the effect of the life course trajectory in BMI from menarche to midlife on age at menopause. Some women choose to take hormone therapy (HT) before they reach menopause often during the perimenopause to relieve menopausal symptoms [35]. Since a lower BMI may be associated with use of HT [36,37], we also consider the association of BMI with age at start of HT use. We take account of the potential confounding variables of parity, cigarette smoking and childhood and adult social class.

## 2 Materials and methods

The MRC National Survey of Health and Development is a socially stratified birth cohort of 2547 women and 2815 men. There have been over 20 follow-ups of the whole cohort between their birth in 1946 and age 53 years [38]. Those interviewed at age 53 years were, in most respects, representative of the British born population of that age [39]. Women cohort members were sent annual postal questionnaires about health during the middle years of life between the ages of 47 and 54 years. At the time of their 57th birthday in 2003, an additional follow-up questionnaire allowed age at menopause to be derived for all women who were postmenopausal by that age. In addition to the postal questionnaires, similar data on menopause were obtained from the home visit at age 53 years. Of the original birth cohort of women 6% had died (*n* = 154) by age 47 years, 9% were living abroad and were not in contact with the study (*n* = 232), 12% had refused to participate (*n* = 296) and 3% could not be traced (*n* = 87). Of the 1778 eligible women, 1735 women returned at least one postal questionnaire or provided information during the home visit. Each data collection received MREC approval and informed consent was obtained from all participants.

Definition of menopausal status (premenopausal, perimenopausal and postmenopausal) was based on the criteria used in the Massachusetts Women's Health Study [14,40]. Date of menopause was defined retrospectively following 12 months of amenorrhea. The date of inception of the perimenopause was defined either as the date of last period if periods had stopped for between 3 and 12 months, or as the date when a woman reported that her menstrual cycle length had become more irregular in the preceding 12 months.

Women experiencing cessation of periods other than by natural menopause were identified from the health questionnaires where they were asked to provide dates of hysterectomy or bilateral oophorectomy operations. A complete monthly record of HT use was also collected. The use of the majority of such preparations cause bleeding so it was not possible to define age at menopause, according to the definitions of menopausal status used here, if HT was started before cessation of menstrual cycles was reported.

Height and weights were measured at 15, 36, and 43 years and self-reported on postal questionnaires at 20 and 26 years. Body mass index (BMI), defined as weight/height^2^, was calculated at each age. A categorical variable was also calculated at each age indicating underweight, normal weight, overweight and obese, using the standard cut points of 20, 25 and 30 kg/m^2^. A trajectory based on BMI at ages 20 and 36 years was also defined. These two time points were chosen as they represent the first measure after all women have reached menarche and the latest measure where all women are still premenopausal. Defining all possible BMI trajectories was not feasible because of small numbers in some cases; hence we defined five groups (always underweight, always obese/overweight, always normal weight, increasing at least one category, and decreasing at least one category). Abdominal and hip circumferences were also measured at age 43 years using standardised protocols, from which waist to hip ratio was calculated.

Potential confounders, identified because of their possible association with age at menopause and with BMI, were parity, smoking and childhood and adult social class. Records of all live births have been collected throughout the adult life of the cohort. Smoking was defined using information given when the cohort were 36 years of age since this represents a premenopausal measure for the entire cohort. Socioeconomic status in childhood was represented by social class assigned from father's occupation when the survey members were aged 4 years. In adulthood, occupational social class, based on the current or most recent occupation at age 43 years was used.

Weight at age 2 years and breast feeding have been shown to be associated with age at menopause in NSHD [41,42] and may also be related to adult BMI as weight tracks across the whole life course and some studies suggest that BMI is associated with infant nutrition [43]. Weight was measured at age 2 years and grouped into fifths. Information on duration of breast feeding was obtained from mother's reports to health visitors when the survey members were 2 years of age, and was here categorised into never breast fed, breast fed for 3 months or less, 4–6 months and 7 months or more.

### 2.1 Statistical methods

Cox's proportional hazard models [44] were used to obtain estimates of the hazard ratio for the risk factors. The small number of women (*n* = 37) whose periods stopped for other surgical reasons (usually endometrial ablation) or medical treatment (for example, chemotherapy) before the menopause, and those for whom data were not complete (*n* = 115) were excluded, leaving 1583 women for analysis. Follow-up time was in months since age 25 years and was until menopause (*n* = 695) or if no date of menopause was available, until the date of the first of the following events: start of HT use (*n* = 431) or hysterectomy (or bilateral oophorectomy) (*n* = 347) or start of OC use (*n* = 3) or last returned questionnaire if premenopausal or perimenopausal at that time (*n* = 107). Follow-up was treated as censored if the first event was not menopause. Where the date of menopause was not specified to the nearest month, the mid-point of the year of menopause was taken.

The assumption of proportional hazards was checked both by inspection of plots and by the use of time-dependent covariates. Initially, unadjusted estimates of the hazard ratio for BMI at each age, both as a continuous measure and as a categorical variable were calculated. We also considered waist and hip circumference and waist to hip ratio at age 43 years in a similar way. Checks for linearity were carried out by adding a quadratic term when considering BMI as a continuous variable and, tests for linear trend across the ordered categorical variables were undertaken. When considering the association with BMI at 43 years, analyses were conducted excluding those who had reached menopause (*n* = 23) or had follow-up censored (*n* = 151) prior to that age. We then tested whether the trajectory variable was associated with age at menopause. The influence of the potential confounders, cigarette smoking, parity and childhood and adult social class was assessed and interactions between all body size measures and cigarette smoking were tested, as an earlier analysis in this cohort suggested that BMI was associated with age at perimenopause in smokers and ex-smokers only [11]. The potential confounders of weight at 2 years and breast feeding were tested in further models.

To investigate the impact of the selective removal of HT users from follow-up a competing risks framework was utilised [45,46]. The start of HT use was considered as an additional event of interest and as a competing risk to natural menopause [47]. Such an approach first involved all models being rerun, using Cox's proportional hazard models as for the main analysis, with start of HT, rather than menopause, as the event of interest. The follow-up times of women who had a known date of menopause were censored at the age menopause was reached. All other follow-up times were treated in the same way as for the main age at menopause analysis. Hence, results for HT use are interpreted in the same way as those for menopause with a hazard ratio greater than 1 representing earlier age at initiation of HT. All analyses were then repeated with the first event (either natural menopause or start of HT) as the outcome of interest. All analyses were carried out using the statistical package SAS [48].

## 3 Results

Mean BMI increased with increasing age. Thirty-four percent of women increased by at least one BMI category between ages 20 and 36 years but only 7% showed a decrease over this period (Table 1). There were no significant overall associations between BMI at any age and age at menopause (Table 2). The strongest overall association was observed with BMI at age 36 years (*p* = 0.2) where the underweight women did have a significantly higher rate of menopause than the normal weight group, or equivalently, an earlier age at menopause. When grouping underweight at 36 years versus the rest a significant association was observed (*p* = 0.03) with a hazard ratio (95% CI) of 1.29 (1.02, 1.64). There was no association with waist or hip circumference or their ratio or the trajectory variable.

After adjustment for parity, cigarette smoking and childhood and adult social class, the association between BMI at 36 years and age at menopause, and the association with the binary variable (underweight versus not underweight) remained the same (results not shown). There was little change in these associations after adjustment for weight at 2 years and breast feeding. There was no evidence of effect modification by cigarette smoking for any of the body size variables (*p* for interaction >0.1 in all cases).

BMI at every age was strongly and linearly associated with age at HT use and BMI from 26 years onwards was associated with age at first event (HT use or menopause) (Table 3). Higher BMI at all ages was associated with older age at initiation of HT use. For first event, there was a similar although weaker linear inverse relationship with BMI at 36 years, but a suggestion of a *U*-shaped relationship at 26 years. The non-linear nature of this relationship was backed up by the significant quadratic term when BMI was modelled as a continuous variable (*p* = 0.04). Both higher waist and hip circumference were associated with later HT use and first event, but waist to hip ratio was not. BMI trajectory between 20 and 36 years was also strongly related with age at HT use with the always underweight group starting HT at a younger age than the normal weight group and the always obese or overweight group using it later (Table 3). A similar, although weaker, association was seen between trajectory and first event.

There was no evidence that the associations between BMI and HT at any age were weakened on addition of parity, cigarette smoking or social class or by weight at 2 years and breast feeding (results not shown). There was also very little confounding with parity, smoking and social class of the associations between BMI at 36 years, abdominal or hip circumference at 43 years or BMI trajectory and first event (menopause or HT use) (Table 4). The relationships were slightly strengthened after adjustment. There was only slight confounding on addition of breast feeding and weight at 2 years.

## 4 Discussion

We found no dose response association between BMI and age at natural menopause or between BMI trajectory from 20 to 36 years and natural menopause, but underweight women at age 36 years did have a significantly earlier menopause than other women. We did find consistent associations between body size and age at start of HT use with lower BMI, lower waist and hip circumference and underweight trajectory being related to earlier start of use.

The lack of association between BMI and age at natural menopause was consistent with the majority of other studies [8–11,14–20]. However many of these studies have relied on self-reported weight, which may be subject to bias, or BMI measured at the same time as assessment of menopausal status or only a few years prior to menopause. The fact that it is only BMI at age 36 years that shows any relationship with age at menopause may be due to BMI at 36 years being the latest premenopause measure for all women and because underweight at this age is an indicator of being thin throughout life. This is consistent with the higher hazard ratios in the women who remained underweight between 20 and 36 years, particularly when compared with those who increased BMI category between 20 and 36 years. Few previous studies have considered BMI or weight in earlier adult life or change in these measures, and most of those that have were limited by recall bias resulting from the use of retrospective self-reported measures of earlier weight [8,18,20,23,29]. Of those that found an association, obesity at age 18 years [23], and large weight gain from age 20 years [8] were related to a later menopause and large weight losses to an earlier menopause [29]. A study from Japan [13] considered repeated measures of BMI, but only from age 39 years onwards, and showed that higher BMI was related to later age at menopause. In the NSHD, we found little evidence that changes in body size across the life course influenced age at menopause, although very few women experienced large decreases in weight between 20 and 36 years and therefore the statistical power to detect an effect for this group was low. Over 50% of women who were in a lower BMI category at 36 years compared with 20 years moved from normal to underweight.

Additional analyses using annual reports of hot flushes (up to age 54 years) as a time-varying covariate suggested that such symptoms were associated strongly with both HT use and natural menopause, with the association being somewhat stronger for HT use. This lends support to the possibility that these women were approaching menopause and thus that start date for HT may be a reasonable proxy measure for age at menopause. In the absence of HT use it is therefore possible that underweight women at 26 and 36 years and those with small waist and hip circumferences would experience an earlier menopause as suggested by the results from the first event (menopause or start of HT) analyses. This was in agreement with a previous analysis of this dataset with follow-up to age 50 years [11] that found a stronger relationship between BMI at 36 years and age at perimenopause, particularly in cigarette smokers, than with age at menopause. This was due to underweight women starting HT during the perimenopausal period. Since, underweight women have lower endogenous estrogen levels than heavier women, it has generally been hypothesised that thin women are more likely to experience vasomotor symptoms [49]. This could potentially explain their greater HT use at any given age. However, studies to date have actually found higher BMI to be related with increased reporting of vasomotor symptoms [49]. Alternatively, underweight women may start HT before they reach the perimenopause because of other behavioural characteristics, possibly relating to concerns regarding risk of osteoporosis or of weight gain [36]. Behavioural factors are a more likely explanation for our findings comparing the obese and overweight groups, who may be less concerned about osteoporosis for example, with the normal weight group. Overweight and obese women had considerably lower rates of HT use prior to menopause compared with normal weight women. However, the directions of these effects were different to those found in the age at menopause analyses where rates were similar or higher.

The associations with both menopause and HT use were unaffected by cigarette smoking, parity or childhood and adult social class. Parity only goes some way to adjusting for subfertility and hence that the findings reflect subfertility in the underweight women remains a possibility. That we found only weak evidence to suggest that underweight women had an earlier menopause than others may be because it is undernutrition over long periods of time which influences age at menopause as opposed to differences in nutrition within well-nourished populations. A study of women in New Guinea found that the median age of menopause in a population who had suffered severe and prolonged malnourishment, and who were of short height and low weight, was 4 years earlier than women in the same region with better nourishment [50]. Weight reduction dieting has also been linked to early menopause in one study [8] and it may be that the women who were underweight at 36 years were those with a history of dieting. The fact that hip as well as waist circumference, but not their ratio, were associated with HT use suggested that HT users might have small skeletal size as well as low fat mass. We previously observed that women of low weight at 2 years had an earlier menopause than others [41,42]. A study of the Dutch famine of 1944–1945 found that women who had been severely exposed to the famine, especially those who had been aged 2–6 years, had an earlier menopause than unexposed women [51]. However, adjusting for weight at 2 years and breast-feeding duration did not have a great impact on the relationship between body size and age at menopause or HT use.

The main advantage of this study is that NSHD is a prospective cohort and so BMI was obtained across the life course allowing a thorough investigation of BMI in early adult life and BMI trajectories and age at menopause. At ages 15, 36 and 43 years heights and weights in NSHD were measured and are thus not subject to recall or reporting bias. Measures at 20 and 26 were self-reported, but were reported at those ages rather than being recalled many years later. Age at menopause was subject to recall bias only for women who reached menopause before the age of 47, when the first postal questionnaire was sent. Around 9% of the women had experienced menopause before age 46. The relatively high rate of use of HT prior to menopause is an important issue to consider in our study and other epidemiological investigations of the natural menopause where reported menstrual change is used as the basis for definition of menopausal status. The analyses presented here assume independence of menopause and start of HT use, which was unlikely to be valid in practice. The results presented in this paper can only be interpreted under the conditions existing in the study and may not be generalisable to populations with different patterns of HT use. Our investigation of the effect of BMI on HT use prior to menopause goes some way to investigating the influence of the selection process. To our knowledge, this is the first study to have examined the effect of BMI during the reproductive years and the start of HT use as well as age at natural menopause. Many previous studies of age at natural menopause have either excluded HT users [8,10,12,18,20] or included them with a menopausal status therefore based on potentially inaccurate reports of last menstrual cycle (often adding HT use as an explanatory variable in the model) [14,16,17,22,29]. Different levels of population HT use and the different treatment of HT users in analyses may have led to the discrepant findings in previous research. Analyses in populations with low rates of HT use would be useful to further investigate associations between underweight and menopause.

In conclusion, there is little evidence that BMI influences age at menopause in a way that is clinically relevant for later disease risk. Low BMI at all ages and underweight trajectory (20–36 years) were related to earlier age at start of HT, which may be a proxy measure for age at menopause. In order to understand the postmenopausal health implications for underweight women, further studies need to assess whether the association is due to an earlier menopause among underweight women or due to behavioural characteristics which mean that they start HT early, independent of menopausal status.

## Conflict of interest

None.

## Figures and Tables

**Table 1:** Summary statistics for BMI across adult life, waist and hip circumference, behavioural and social factors for women

Variable	*N* (%)	Mean (S.D.)
Follow-up event^a^	1583	
Menopause	695 (43.9%)	
HT use	431 (27.2%)	
Hysterectomy	347 (21.9%)	
OC use	3 (0.2%)	
Pre/perimenopause at last returned questionnaire	107 (6.8%)	

BMI at 15 years (kg/m^2^)	1191	20.6 (3.0)
BMI at 20 years (kg/m^2^)	1285	21.9 (3.0)
BMI at 26 years (kg/m^2^)	1372	22.4 (3.3)
BMI at 36 years (kg/m^2^)	1405	23.6 (4.0)
BMI at 43 years (kg/m^2^)	1436	25.2 (4.8)
Waist circumference at 43 years (cm)	1437	77.6 (11.2)
Hip circumference at 43 years (cm)	1433	100.5 (10.0)
Waist to hip ratio at 43 years (cm)	1430	77.2 (6.3)

BMI trajectory 20–36 years	1162	
Underweight	100 (8.6%)	
Normal weight	482 (41.5%)	
Decrease category	84 (7.2%)	
Increase category	396 (34.1%)	
Overweight/obese	100 (8.6%)	

Parity	1447	
Nulliparous	176 (12.2%)	
1 child	182 (12.6%)	
2 children	653 (45.1%)	
3 children	314 (21.7%)	
4+ children	122 (8.4%)	

Cigarette smoking at 36 years	1417	
Non-smoker	490 (34.6%)	
Ex-smoker	482 (34.0%)	
Smoker	445(31.4%)	

Childhood social class	1392	
Non-manual	601 (43.2%)	
Manual	791 (56.8%)	

Adult social class	1326	
I/II	473 (35.7%)	
III (non-manual)	482 (36.3%)	
III (manual)	117 (8.8%)	
IV/V	254 (19.2%)	

a Follow-up was until menopause or if no date of menopause was available, until the first of the following events: start of HT use, hysterectomy, start of OC use, last returned questionnaire if premenopausal or perimenopausal at that time.

**Table 2:** Unadjusted hazard ratios for menopause by BMI across adult life and waist and hip circumference at 43 years obtained from Cox's proportional hazard models

	*N* (events)	Hazard ratio	(95% CI)	*p*-Value
BMI at 15 years	1191 (521)			
Per kg/m^2^		1.01	(0.98, 1.04)	0.6
Underweight	551 (238)	1.03	(0.86, 1.23)	0.6
Normal	556 (238)	1		
Overweight/obese	84 (45)	1.14	(0.83, 1.57)	

BMI at 20 years	1285 (570)			
Per kg/m^2^		1.01	(0.98, 1.03)	0.6
Underweight	340 (145)	0.99	(0.82, 1.21)	0.9
Normal	798 (349)	1		
Overweight	119 (59)	1.00	(0.76, 1.32)	
Obese	28 (17)	1.17	(0.72, 1.90)	

BMI at 26 years	1372 (616)			
Per kg/m^2^		1.00	(0.98, 1.03)	0.9
Underweight	283 (123)	1.07	(0.88, 1.32)	0.6
Normal	863 (386)	1		
Overweight	185 (82)	0.97	(0.76, 1.23)	
Obese	41 (25)	1.26	(0.84, 1.89)	

BMI at 36 years	1405 (620)			
Per kg/m^2^		0.99	(0.98, 1.01)	0.6
Underweight	185 (81)	1.30	(1.02, 1.65)	0.2
Normal	858 (372)	1		
Overweight	261 (116)	0.99	(0.81, 1.23)	
Obese	101 (51)	1.05	(0.78, 1.41)	

BMI at 43 years^a^	1262 (604)			
Per kg/m^2^		0.99	(0.98, 1.01)	0.4
Underweight	81 (44)	1.16	(0.84, 1.59)	0.5
Normal	698 (326)	1		
Overweight	324 (151)	0. 91	(0.75, 1.10)	
Obese	159 (83)	1.02	(0.80, 1.29)	

At 43 years^a^				
Waist (per 10 cm)	1263 (605)	0.96	(0.90, 1.04)	0.3
Hip (per 10 cm)	1259 (602)	0.94	(0.88, 1.03)	0.2
Waist:hip ratio (per %)	1256 (601)	1.00	(0.99, 1.01)	0.8

Trajectory 20–36 years	1162 (519)			
Underweight	100 (42)	1.18	(0.85, 1.65)	0.2
Normal weight	482 (215)	1		
Decrease category	84 (40)	1.19	(0.85, 1.66)	
Increase category	396 (169)	0.86	(0.70, 1.05)	
Overweight/obese	100 (53)	0.96	(0.71, 1.30)	

a Excludes those with follow-up of 43 years or less.

**Table 3:** Unadjusted hazard ratios from a competing risks analysis with HT and first event (menopause or HT) as events of interest

	HT				First event			
	*N* (events)	Hazard ratio	(95% CI)	*p*-Value	*N* (events)	Hazard ratio	(95% CI)	*p*-Value
BMI at 15 years	1191 (340)				1191 (861)			
Per kg/m^2^		0.94	(0.91, 0.98)	0.004		0.98	(0.96, 1.01)	0.2
Underweight	551 (174)	1.14	(0.92, 1.42)	0.02	551 (412)	1.07	(0.93, 1.23)	0.4
Normal	556 (152)	1			556 (390)	1		
Overweight/obese	84 (14)	0.57	(0.33, 0.98)		84 (59)	0.92	(0.70, 1.21)	

BMI at 20 years	1285 (351)				1285 (921)			
Per kg/m^2^		0.95	(0.91, 0.98)	0.006		0.99	(0.96, 1.01)	0.2
Underweight	340 (108)	1.13	(0.90, 1.42)	0.004	340 (253)	1.05	(0.90, 1.22)	0.2
Normal	798 (220)	1			798 (569)	1		
Overweight	119 (18)	0.52	(0.32, 0.84)		119 (77)	0.82	(0.65, 1.04)	
Obese	28 (5)	0.55	(0.23, 1.32)		28 (22)	0.93	(0.60, 1.42)	

BMI at 26 years	1372 (368)				1372 (984)			
Per kg/m^2^		0.96	(0.93, 0.99)	0.01		0.99	(0.97, 1.01)	0.1
Underweight	283 (98)	1.35	(1.07, 1.72)	0.002	283 (221)	1.18	(1.02, 1.38)	0.05
Normal	863 (227)	1			863 (613)	1		
Overweight	185 (36)	0.71	(0.50, 1.02)		185 (118)	0.87	(0.72, 1.06)	
Obese	41 (7)	0.57	(0.27, 1.21)		41 (32)	1.00	(0.70, 1.42)	

BMI at 36 years	1405 (382)				1405 (1002)			
Per kg/m^2^		0.94	(0.92, 0.97)	<0.001		0.98	(0.96, 0.99)	0.003
Underweight	185 (81)	1.31	(1.00, 1.72)	<0.001	185 (145)	1.31	(1.09, 1.57)	0.001
Normal	858 (372)	1			858 (626)	1		
Overweight	261 (116)	0.61	(0.44, 0.83)		261 (161)	0.84	(0.71, 1.00)	
Obese	101 (51)	0.58	(0.36, 0.92)		101 (70)	0.86	(0.67, 1.11)	

BMI at 43 years^a^	1262 (389)				1262 (993)			
Per kg/m^2^		0.96	(0.94, 0.99)	0.001		0.98	(0.97, 1.00)	0.008
Underweight	81 (44)	0.93	(0.62, 1.38)	0.005	81 (71)	1.06	(0.83, 1.36)	0.09
Normal	698 (326)	1			698 (561)	1		
Overweight	324 (151)	0.85	(0.67, 1.08)		324 (248)	0.88	(0.76, 1.02)	
Obese	159 (83)	0.53	(0.36, 0.78)		159 (113)	0.82	(0.67, 1.00)	

At 43 years^a^								
Waist (per 10 cm)	1264 (389)	0.88	(0.80, 0.97)	0.009	1263 (994)	0.93	(0.88, 0.99)	0.01
Hip (per 10 cm)	1260 (388)	0.84	(0.76, 0.94)	0.002	1259 (990)	0.91	(0.85, 0.97)	0.003
Waist:hip ratio (per %)	1257 (386)	0.99	(0.98, 1.01)	0.4	1256 (987)	1.00	(0.99, 1.01)	0.5

Trajectory 20–36 years	1162 (313)				1162 (832)			
Underweight	100 (37)	1.34	(0.93, 1.93)	0.002	100 (79)	1.26	(0.99, 1.61)	0.006
Normal weight	482 (139)	1			482 (354)	1		
Decrease category	84 (21)	0.95	(0.60, 1.50)		84 (61)	1.09	(0.83, 1.44)	
Increase category	396 (103)	0.86	(0.66, 1.11)		396 (272)	0.86	(0.73, 1.00)	
Overweight/obese	100 (13)	0.41	(0.23, 0.72)		100 (66)	0.76	(0.58, 0.98)	

a Excludes those with follow-up of 43 years or less.

**Table 4:** Hazard ratios for first event (menopause or HT) by BMI at 36 years, waist and hip circumference at 43 years and BMI trajectory (20–36 years)

		Unadjusted			Adjusted for parity, cigarette smoking, childhood and adult social class		
	*N* (events)	Hazard ratio	(95% CI)	*p*-Value	Hazard ratio	(95% CI)	*p*-Value
BMI at 36 years	1170 (851)						
Per kg/m^2^		0.98	(0.96, 1.00)	0.03	0.98	(0.96, 1.00)	0.02
Underweight		1.30	(1.07, 1.58)	0.01	1.32	(1.08, 1.61)	0.005
Normal		1			1		
Overweight		0.87	(0.72, 1.05)		0.86	(0.71, 1.03)	
Obese		0.90	(0.68, 1.18)		0.86	(0.65, 1.15)	

Underweight		1.35	(1.11, 1.63)	0.004	1.37	(1.13, 1.67)	0.002
Not underweight		1			1		

At 43 years^a^							
Waist (per 10 cm)	1039 (830)	0.93	(0.87, 0.99)	0.02	0.92	(0.86, 0.98)	0.01
Hip (per 10 cm)	1035 (826)	0.91	(0.85, 0.98)	0.008	0.90	(0.84, 0.97)	0.006

Trajectory 20–36 years	1008 (731)						
Underweight		1.27	(0.98, 1.65)	0.05	1.29	(1.00, 1.68)	0.03
Normal weight		1			1		
Decrease category		1.08	(0.81, 1.45)		1.02	(0.76, 1.37)	
Increase category		0.87	(0.74, 1.03)		0.86	(0.72, 1.02)	
Overweight/obese		0.84	(0.63, 1.12)		0.78	(0.58, 1.05)	

Results from unadjusted models and models adjusted for behavioural and social factors.

a Excludes those with follow-up of 43 years or less.
